# Commentary on: A 10-Year Review of Surgical Outcomes at a Resident Aesthetic Clinic

**DOI:** 10.1093/asjof/ojac080

**Published:** 2022-11-03

**Authors:** Kiya Movassaghi, Alexander Jacob Gougoutas

**Affiliations:** Clinical assistant professor, Department of Plastic Surgery, Oregon Health and Science University, Eugene, OR, USA; Aesthetic fellow in private practice, Eugene, OR, USA

This original article^1^ demonstrates that resident aesthetic clinics, or RACs, provide residents with the skills necessary to safely perform many common cosmetic procedures—a finding demonstrated across multiple academic institutions^2–4^ and mirrored by my experiences as an aesthetic fellowship director. Though safety is a critical component of all surgical training, the uniquely subjective nature of aesthetic surgery makes it an insufficient metric for evaluating the competency of future aesthetic surgeons. To date, there is a paucity of studies examining subjective clinical outcomes arising from RACs.^5^ Such outcome studies should be encouraged as they are invaluable in shaping aesthetic education (Video).

Aesthetic surgery is an exceedingly diverse discipline, the demand for which is only increasing.^6^ As this demand grows, so too will the number of nonplastic surgeons who offer cosmetic services. As aesthetic surgeons, we must embrace all procedures our specialty has to offer, whether surgical or nonsurgical. A dedicated aesthetic fellowship should be encouraged by program directors and strongly considered by those residents hoping to incorporate aesthetic surgery into their future practices. Additional focused training will increase confidence in performing the full spectrum of aesthetic surgery procedures and assure our continued ownership of this discipline.

## Supplementary Material

ojac080_Supplementary_Data
