# Supplementary material for: Commentary on: A 10-Year Review of Surgical Outcomes at a Resident Aesthetic Clinic
Source: Aesthet Surg J Open Forum. 2022 Nov 3;4:ojac080. doi: 10.1093/asjof/ojac080 (PMC9897167; doi:10.1093/asjof/ojac080)
Supplement: ojac080_Supplementary_Data [file ojac080_Supplementary_Data.docx]

Video Transcript

I want to applaud the authors of this original article entitled "A 10-year Review of Surgical Outcomes at a Resident Aesthetic Clinic”. Despite the limitations inherent to its retrospective study design, I believe this article accomplishes its stated objectives, and is a valuable contribution to a growing body of research demonstrating both the utility and overall safety of resident aesthetic clinics, or RACs.

Cosmetic surgery is distinct from all other plastic surgery sub-specialties. Whereas reconstructive surgery measures its success in the *restoration* of rudimentary form and function, successful cosmetic surgery necessitates subjective *refinement* of disharmonious features.

It is for these reasons that aesthetic surgery education poses a particular challenge for providers and residents alike.

My experience as an aesthetic fellowship director echo many of this article’s conclusions and discussion points. Recent graduates do demonstrate more comfort with breast and body procedures than they do with the more nuanced and technically demanding facial aesthetics, particularly rhinoplasty and lower eyelid surgeries. Across most patient populations however, fellows are consistently able to identify and minimize patient specific risk factors and tailor operative planning accordingly. In the operating room they demonstrate sound fundamental technique and awareness of common pitfalls. They can competently develop and execute plans to manage post-operative complications.

In this sense they are indeed “safe”.

I would stress, however, that safety, while imperative, hardly equates with competency. Aesthetic surgery is a visual art, and one that largely measures its success through patient-perceived outcomes. As such, assessing the value of an RAC as an effective tool in training competent plastic surgeons must focus on long term clinical results. As the authors themselves point out, their article falls short in this regard. To this point, a recent literature review from the University of Pennsylvania identified 415 articles pertaining to RACs. Of these less than 3% addressed patient outcomes.

I’d like to end by saying that cosmetic surgery is an exceedingly diverse and evolving discipline. Novel techniques and technologies are ever-present, ensuring the continued growth of our specialty. While inspiring, these trends also deserve a note of caution. I am increasingly concerned by the growing number of inadequately trained providers offering aesthetic services. It is my firm belief that we as cosmetic surgeons must continue to embrace the full spectrum of both operative and non-operative procedures we’ve developed. The desire and confidence to do so beings with education.

It is for this reason I would strongly encourage an aesthetic fellowship. This additional year of focused training will grant you the insights and exposure necessary to tackle all aesthetic endeavors- from the most rudimentary, primary procedures, to the most complex revisionary dilemmas.
